# Fabrication of SiO_2_/PEGDA Inverse Opal Photonic Crystal with Fluorescence Enhancement Effects

**DOI:** 10.1155/2021/6613154

**Published:** 2021-02-23

**Authors:** Van-Phuc Dinh, Duy-Khoi Nguyen, Quang-Hung Nguyen, Thi-Thuy Luu, Thi Hai Yen Pham, Thi Thu Ha Vu, Han-Sheng Chuang, Hong-Phong Pham

**Affiliations:** ^1^Future Materials & Devices Laboratory, Institute of Fundamental and Applied Sciences, Duy Tan University, Ho Chi Minh City 700000, Vietnam; ^2^Faculty of Environmental and Chemical Engineering, Duy Tan University, Da Nang 550000, Vietnam; ^3^Institute of Fundamental and Applied Sciences, Duy Tan University, Ho Chi Minh City 700000, Vietnam; ^4^Institute of Chemistry, Vietnam Academy of Science and Technology, 18-Hoang Quoc Viet Road, Cau Giay, Ha Noi, Vietnam; ^5^Department of Biomedical Engineering, National Cheng Kung University, Tainan 701, Taiwan; ^6^Graduate University of Science and Technology, 18- Hoang Quoc Viet Road, Cau Giay, Ha Noi, Vietnam

## Abstract

The present paper reports the fabrication of inverse opal photonic crystals (IOPCs) by using SiO_2_ spherical particles with a diameter of 300 nm as an opal photonic crystal template and poly(ethylene glycol) diacrylate (PEGDA) as an inverse opal material. Characteristics and fluorescence properties of the fabricated IOPCs were investigated by using the Fourier transform infrared spectroscopy (FTIR), scanning electron microscopy (SEM), X-ray diffraction (XRD), reflection spectroscopy, and fluorescence microscopy. The results clearly showed that the IOPCs were formed comprising of air spheres with a diameter of ∼270 nm. The decrease in size led to a decrease in the average refractive indexes from 1.40 to 1.12, and a remarkable stopband blue shift for the IOPCs was thus achieved. In addition, the obtained results also showed a fluorescence enhancement over 7.7-fold for the Fluor® 488 dye infiltrated onto the IOPCs sample in comparison with onto the control sample.

## 1. Introduction

Photonic crystals (PhC) with a periodic structure have significant effects on the propagation of electromagnetic (EM) waves due to the diffraction of photons in a limited wavelength range from the lattice planes [1, 2], leading to the allowance or restriction of the propagation of EM waves through the material structure. When the EM radiation is forbidden in the wavelength region, it cannot be transmitted, resulting in the reflection from the crystal lattice, known as Bragg diffraction [3]. As the result, the photonic bandgap (PBG) is formed. The changes in the refractive index of PhC lead to the adjustment of the PBG wavelengths and reflection intensity. Consequently, the photonic especially fluorescent properties of the materials are changed [4, 5].

SiO_2_ material with a porous structure, known as a stably, nontoxically, harmlessly and low-costly photonic crystalline material, has been widely applied to various areas, such as catalyst supports [6–8], adsorbents [9–11], chromatographic materials [12, 13], and biosensors [14, 15]. In particular, the SiO_2_ inverse opal photonic crystal (IOPC) biosensors have been fabricated based on changes in their photoluminescence properties or reflective spectra. It has been shown in the literature [16] that the IOPCs can change not only the wavelengths of PBGs but also enhance the fluorescent intensity. Materials used as the inverse opal materials for fabrication of IOPCs are varieties of hydrogels, such as polystyrene [5, 17], poly(4-vinylbenzyl chloride-co-methyl methacrylate) [18], and poly(styrene-co-methyl methacrylate-co-acrylic acid) [19]. Although poly(ethylene glycol) diacrylate (PEGDA) is a derivative of polyethylene glycol and has been widely used for various biomedical applications due to its biocompatibility [20] and easy excretion from the body [21], the use of this hydrogel as an inverse opal material in IOPCs has still been limited. For instance, studies of Park et al. focused on the fabrication of humidity sensors and biosensors for the detection of immunoglobulin G [22, 23]. Though the obtained results clearly show the change in colour of the fabricated IOPCs with the presence of detection targets, the improvement of fluorescence intensity of IOPCs is still required to enhance the sensitivity of target detections.

In order to fabricate the IOPCs, two techniques have been widely used, namely, thermal degradation [5, 17–19, 24] and chemical etching [25, 26]. The thermal degradation has been commonly used for the treatment of organic templates, while the chemical etching has been regularly applied to remove the inorganic templates, such as TiO_2_, ZnO, and SiO_2_. Among the chemical etchants, the buffered oxide etch (BOE), a mixture of hydrofluoric acid (HF) and ammonium fluoride (NH_4_F), is often used for etching silicon dioxide on the silicon wafers. In this work, SiO_2_ spherical particles are used as an opal photonic crystal material, whereas PEGDA is utilized as an inverse opal material to fabricate the IOPCs having two-dimensional periodical and microporous structure with the lattice spacing on the order of the wavelength of light. These IOPCs thus not only induce a stopband shift but also exhibit a fluorescence enhancement for Alexa Fluor® 488 dye, which is a green fluorophore and has been commonly used in applications such as immunolabeling, fluorescence microscopy, and flow cytometry.

## 2. Materials and Methods

### 2.1. Chemicals

Nonfunctionalized silica microspheres with a diameter of 300 nm were purchased from the Polyscience Asia Pacific Inc. Poly(ethylene glycol) diacrylate (Mn = 250), ethanol 95%, 2-hydroxy-2-methylpropiophenone 97% (Irgacure 1173), donkey anti-rabbit IgG (H + L), Alexa Fluor® 488 conjugate (A-21206, Invitrogen), and buffered oxide etchant (BOE) were supplied by Sigma-Aldrich. Irgacure 1173, a photoinitiator, was used in radiation curing in the polymerization of PEGDA.

### 2.2. Protocol for IOPC Fabrication

**Scheme 1:**
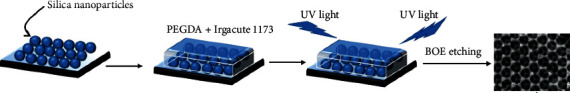
Protocol for the fabrication of IOPCs.

The fabrication of IOPCs was performed following Scheme 1 [22, 23]. Herein, 0.5 mL silica sol suspension was dropped onto a microscope slide after covering with a hydrophobic thin layer and dried in air for 1 day at room temperature. The mixture of 99 wt% PEGDA and 1 wt% 2-hydroxy-2-methylpropiophenone was then added into the dried SiO_2_ for 5 minutes and exposed under the UV light (312 nm) for 5 minutes for the polymerization process. Finally, the chip was etched by BOE for few hours to obtain the IOPCs before being washed by pure water several times.

### 2.3. Instruments

The spectrometer processor (Ocean Optics QE Pro-FL), which has a wavelength range of 350 nm–1100 nm, in conjunction with a halogen light source (Ocean Optics HL-2000) was used to determine the reflected spectra. The fluorescent images of the samples were measured using a fluorescent microscope (BX51, Olympus), whereas the fluorescent intensity was analysed by the ImageJ software. Scanning electron microscopy (SEM, Hitachi S-4800; acceleration of 15–20 kV and working electrode distance of 4–5 mm) was used to characterize the surface morphology. Fourier transform infrared spectroscopy (FTIR, FTIR-PerkinElmer Spectrum 10.5.2) measurements were recorded at the atmospheric pressure with a resolution of 4 cm^−1^ using p-polarized radiation. X-ray diffraction data were collected using Bruker D8 (20 kV, 5 mA) equipped with a LynxEye detector and a conventional Cu anode. Diffractograms were collected at a step of 0.25 s.

## 3. Results and Discussion

### 3.1. Characterizations of PEGDA/SiO_2_ and IOPCs

#### 3.1.1. FT-IR Spectrum

**Figure 1:**
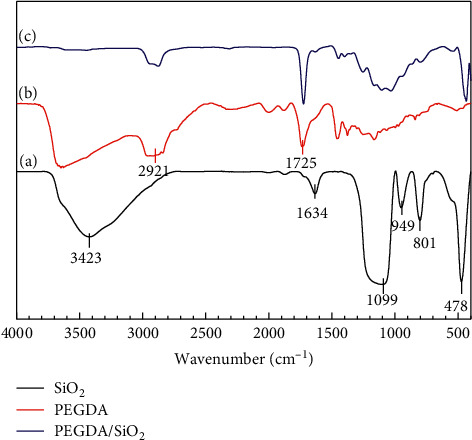
FT-IR spectra of SiO_2_ (curve (a)), PEGDA (curve (b)), and PEGDA/SiO_2_ (curve (c)).

The interactions between PEGDA and SiO_2_ are determined via the FT-IR analysis.  Figure 1 shows the FT-IR spectra of SiO_2_, PEGDA, and PEGDA/SiO_2_. The characteristic peaks belonging to SiO_2_ are clearly observed in curve (a). The bending vibration of the Si–O group is recorded at the wavenumber of 478 cm^−1^, while the peak at 949 cm^−1^ is assigned to the vibration of Si–OH. The peaks at 801 cm^−1^ and 1099 cm^−1^ are related to the vibration of the Si–O–Si group. The vibrations of –OH groups are found at 1634 cm^−1^ and 3423 cm^−1^ [27, 28]. The FT-IR spectrum of PEGDA (curve (b)) shows a typical peak of the carbonyl group (C=O) at the wavelength of 1724 cm^−1^, whereas the peak at the wavelength of 2921 cm^−1^ is attributed to the CH_2_ group of PEGDA [29]. The spectrum of PEGDA/SiO_2_ (curve (c)) shows all dominant peaks corresponding to both PEGDA and SiO_2._ Noticeably, those are related to the methylene group (CH_2_), C=O, and Si-O functional surface groups. These results clearly indicate that PEGDA particles have been successfully grafted to SiO_2_ spheres via physical interactions [27, 30].

#### 3.1.2. SEM Images

**Figure 2:**
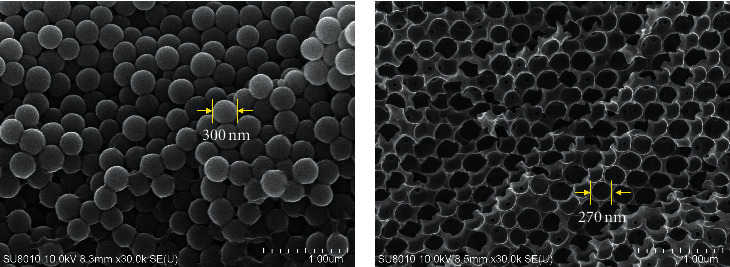
SEM images of PEGDA/SiO_2_ before (a) and after etching with BOE (b).

As seen in Figure 2(a), PEGDA/SiO_2_ template comprises many SiO_2_ spherical particles with a diameter of 300 nm, which are covered by a PEGDA thin film. After etching, the inverse opal photonic structure is created, which contains a large number of micropores with a diameter of ∼270 nm, as seen in Figure 2(b). It is obvious that the diameter of air spherical is shrunk, leading to the variation of the wavelength of the reflected light due to effects on the propagation of electromagnetic waves in a limited frequency range, as discussed below.

#### 3.1.3. XRD Analysis

XRD measurement was performed to study the characteristics of PEGDA/SiO_2_ before and after etching with BOE. The obtained XRD results for SiO_2_, PEGDA/SiO_2_ template, and the IOPCs showed only one peak at 2*θ* = 22° (see supplemental information (available here)). The appearance of this peak is a feature of the amorphous state of silica materials [31] and indicates that the fabrication of IOPCs is not influenced by the etching process.

### 3.2. Reflectance Spectra

The wavelength of reflection peaks (*λ*) can be estimated based on Bragg's equation as follows [32]:

(1) $$\begin{array}{c} \lambda =1.633\;dn_{\text{average}}, \end{array}$$

where *d* (nm) is the center-to-center distance between two neighbouring mesopores and *n*_average_ is the average refractive index of the studied materials. According to the product specification sheet, the refractive indexes of SiO_2_ particles and PEGDA materials are about 1.378 and 1.463, respectively. The average refractive index of the PEGDA/SiO_2_ and IOPCs in the air can be calculated using the following equation [32, 33]:

(2) $$\begin{array}{c} n^{2}=\left(1-\Phi \right)n_{1}^{2}+\;\Phi n_{2}^{2}, \end{array}$$

where *n*_1_ is the refractive index of colloidal crystal, *n*_2_ is the refractive index of the surrounding environment, and Φ is the void ratio of colloidal crystal.

**Figure 3:**
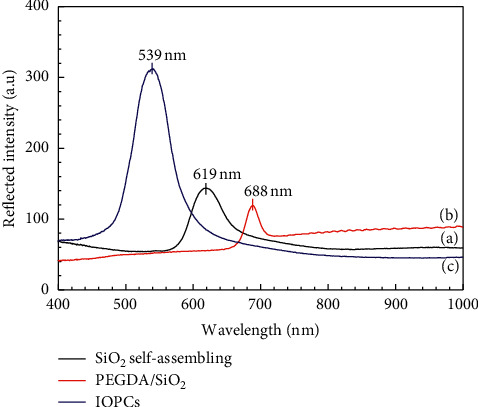
Reflection spectra for SiO_2_ self-assembling (curve (a)), PEGDA/SiO_2_ (curve (b)), and the IOPCs (curve (c)).

The calculated average refractive indexes of the PEGDA/SiO_2_ and the IOPCs were 1.40 and 1.12, respectively. Obviously, the refractive index of the PEGDA/SiO_2_ is decreased after etching because of the replacement of SiO_2_ spheres in the template by voids. In addition, the wavelengths of reflection peaks of SiO_2_, PEGDA/SiO_2_, and the IOPCs calculated from equation (1) are 632, 686, and 548, respectively, which are slightly different from the position of reflection peaks at 619, 688, and 539 nm, respectively, as shown in Figure 3.

**Figure 4:**
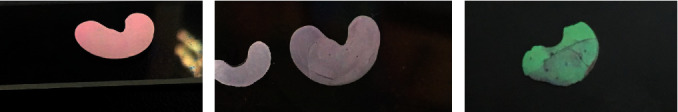
Photographs of SiO_2_ self-assembling (a), PEGDA/SiO_2_ (b), and the IOPCs (c).

This difference is due to the decrease in the diameter of micropores as seen in the SEM images. Thus, there is a good agreement between the wavelengths of reflection peaks shown in the reflection spectra and those calculated by Bragg's equation. The reflection spectra shown in Figure 3 exhibit a remarkable stopband blue shift for the IOPCs when the average refractive index decreases and there is a decrease in the diameter of micropores. This can be clearly observed in images shown in Figure 4, in which the observed colour of SiO_2_ self-assembling, PEGDA/SiO_2_, and the IOPCs is changed from the red colour to orange and green colours correspond to the wavelengths of their photonic stopbands at 688 nm, 619 nm, and 539 nm, respectively. These colour changes exhibit how PhCs made of different materials modify the propagation of light through the photonic stopband. These obtained results are similar to those reported by Subramania and coworkers [34], in which the authors have shown a systematic shift of the reflection peak to longer wavelengths with increasing the diameter of spheres.

### 3.3. Fluorescent Properties

**Figure 5:**
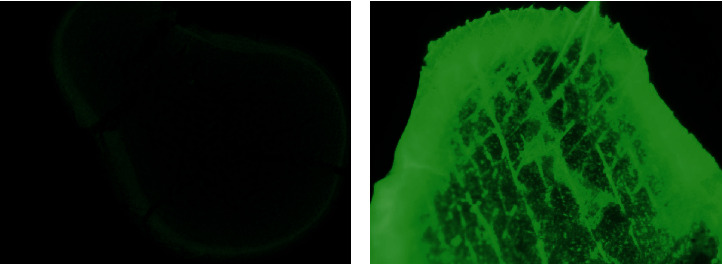
Fluorescence images of PEGDA/SiO_2_ (a) and the IOPCs, for Fluor® 488 dye (b).

**Figure 6:**
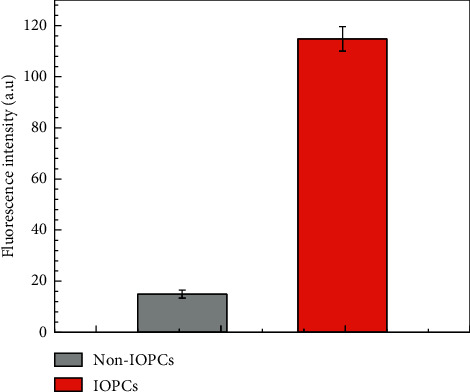
Fluorescence intensities of PEGDA/SiO_2_ (non-IOPCs) and the IOPCs for Fluor® 488 dye.

In order to investigate the effects of the IOPCs on the fluorescence enhancement for Fluor® 488 dye, an accurate amount of 1.0 mg/mL this dye was dropped onto the control PEGDA/SiO_2_ template and the IOPCs sample. The Fluor® 488 dye, which is a bright and green-fluorescent dye, has an absorption peak at a wavelength of 496 nm and a maximum emission around 519 nm. Figures 5 and 6 compare the fluorescence intensity of Fluor® 488 dye infiltrated onto the PEGDA/SiO_2_ template (non-IOPCs) and the IOPCs sample. The experimental data clearly indicate that over 7.7-fold fluorescence enhancement was achieved for Fluor® 488 dye infiltrated onto the IOPCs sample in comparison with onto the control template. The fluorescence enhancement occurs owing to the fact that the stopband of the IOPCs (539 nm) overlaps the emission of fluorescent Fluor® 488 dye (519 nm), leading to a local resonance mode for the propagation of emission.

In the context that many studies have been attempting to improve the fluorescence enhancement of PhCs, such as studies of the introduction of dyes into the PhCs by different methods [35], effects of lattice period, resonant mode polarization, and symmetry on the enhancement effect of PhCs comprised of colloidal quantum dots [36], the resonant interactions between the localized surface plasmon of gold nanoparticles and the emission of the dye infiltrated into the PhCs [37], or the formation of host-guest complexes between Rhodamine B and cucurbituril [38], our obtained results demonstrate for the first time the fluorescence enhancement of Fluor® 488 dye infiltrated onto the IOPCs which is made of PEGDA/SiO_2_. In addition, Fluor® 488 dye is one of the Alexa Fluor dyes, which can be conjugated directly to primary antibodies or to secondary antibodies to amplify signal and sensitivity, since we believe the results of this work can be extended to studies in the field of biological system that might benefit from the added sensitivity afforded by this approach.

## 4. Conclusions

The inverse opal photonic crystals (IOPCs) fabricated by etching the SiO_2_ spheres from the template composed of poly(ethylene glycol) diacrylate (PEGDA) and SiO_2_ within this thin layer (PEGDA/SiO_2_) have been fabricated. This process induced a reduction of air sphere diameter lowering to ∼270 nm. This led to a decrease of the average refractive index of the fabricated IOPCs from 1.40 to 1.12. Consequently, a blue shift of the stopband was achieved. In addition, the IOPCs exhibited a significant enhancement for Fluor® 488 dye up to 7.7-fold in comparison with the control PEGDA/SiO_2_ template. Such obtained results can be helpful for studies in the field of biological system to improve the sensitivity of sensing.

## Data Availability

All the data and supporting materials are included within the article.

## Supplementary Materials

Supplementary Materials Supp. 1: XRD patterns of SiO_2_ (a), PEGDA/SiO_2_ (b), and the IOPCs (c).
