# Supplementary figures and images for: Fabrication of SiO2/PEGDA Inverse Opal Photonic Crystal with Fluorescence Enhancement Effects
Source: J Anal Methods Chem. 2021 Feb 23;2021:6613154. doi: 10.1155/2021/6613154 (PMC7932782; doi:10.1155/2021/6613154)

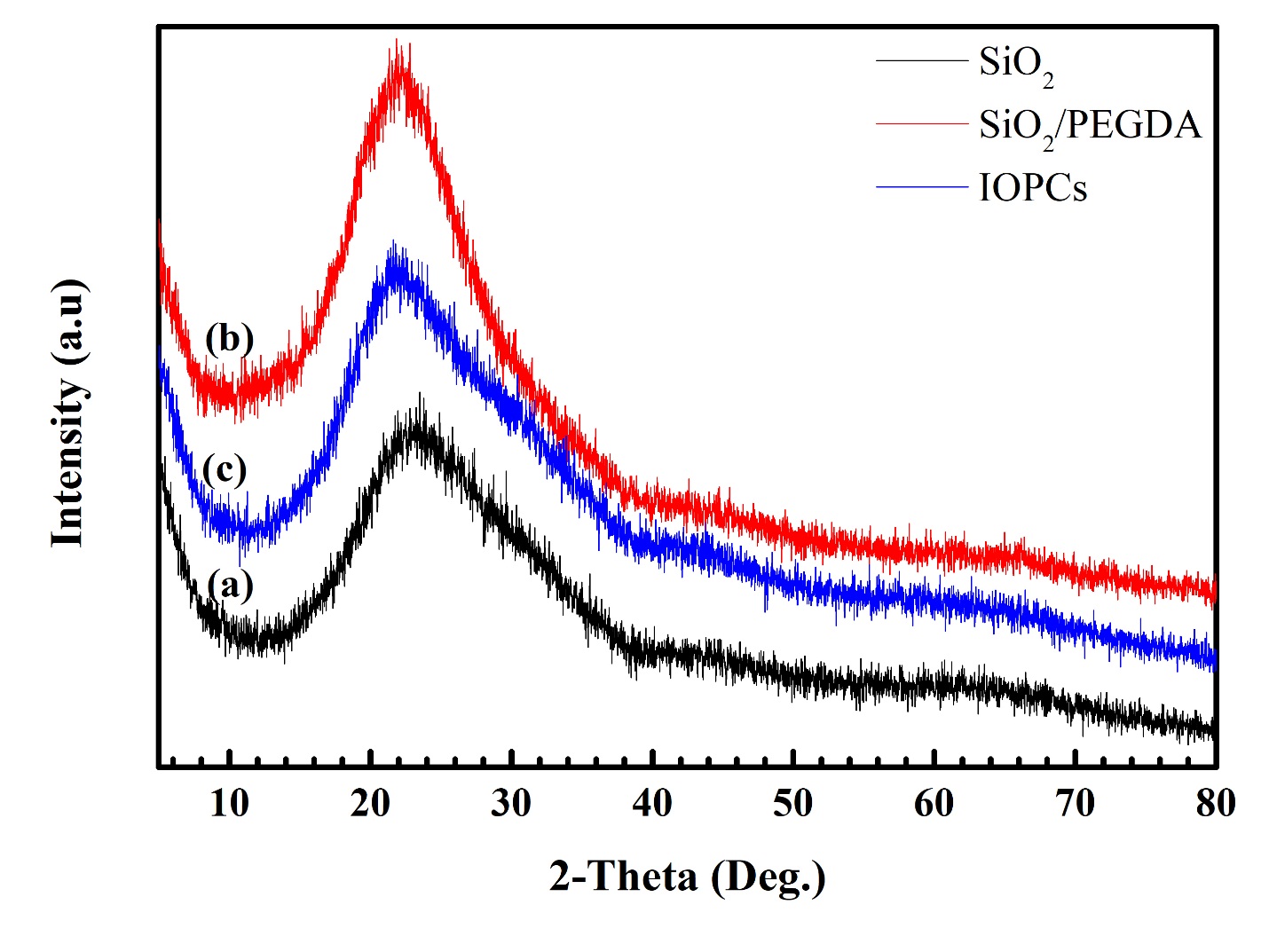


**Supp. 1. XRD** **patterns of SiO2 (a), PEGDA/SiO2 (b) and the IOPCs (c)**

Supplement: Supplementary Materials — Supp. 1: XRD patterns of SiO2 (a), PEGDA/SiO2 (b), and the IOPCs (c). [file 6613154.f1.docx]
